# Harnessing a knowledge translation framework to implement an undergraduate medical education intervention: A longitudinal study

**DOI:** 10.1007/s40037-022-00735-7

**Published:** 2022-12-07

**Authors:** Martine Chamberland, Jean Setrakian, Linda Bergeron, Lara Varpio, Christina St-Onge, Aliki Thomas

**Affiliations:** 1 Department of Medicine, Université de Sherbrooke, Sherbrooke, Quebec Canada; 2 Center for Health Sciences Education, Université de Sherbrooke, Sherbrooke, Quebec Canada; 3 Center for Health Professions Education, Uniformed Services University of the Health Sciences, Bethesda, MD USA; 4 School of Physical and Occupational Therapy and Institute of Health Sciences Education, Faculty of Medicine, McGill University, Montréal, Quebec Canada; 5 Centre for Interdisciplinary Research in Rehabilitation of Greater Montréal, Montréal, Quebec Canada

**Keywords:** Clinical reasoning, Evidence-informed educational intervention, Implementation, Knowledge translation

## Abstract

**Introduction:**

Implementation of evidence-informed educational interventions (EEI) involves applying and adapting theoretical and scientific knowledge to a specific context. Knowledge translation (KT) approaches can both facilitate and structure the process. The purpose of this paper is to describe lessons learned from applying a KT approach to help implement an EEI for clinical reasoning in medical students.

**Methods:**

Using the Knowledge to Action framework, we designed and implemented an EEI intended to support the development of students’ clinical reasoning skills in a renewed medical curriculum. Using mixed-methods design, we monitored students’ engagement with the EEI longitudinally through a platform log; we conducted focus groups with students and stakeholders, and observed the unfolding of the implementation and its continuation. Data are reported according to six implementation outcomes: Fidelity, Feasibility, Appropriateness, Acceptability, Adoption, and Penetration.

**Results:**

Students spent a mean of 24 min on the activity (fidelity outcome) with a high completion rate (between 75% and 95%; feasibility outcome) of the entire activity each time it was done. Focus group data from students and stakeholders suggest that the activity was acceptable, appropriate, feasible, adopted and well-integrated into the curriculum.

**Discussion:**

Through the process we observed the importance of having a structuring framework, of working closely and deliberatively with stakeholders and students, of building upon concurrent evaluations in order to adapt iteratively the EEI to the local context and, while taking students’ needs into consideration, of upholding the EEI’s core educational principles.

**Supplementary Information:**

The online version of this article (10.1007/s40037-022-00735-7) contains supplementary material, which is available to authorized users.

## Introduction

It can be challenging for educators to successfully implement and evaluate new evidence-informed educational interventions (EEI) that require tailoring to their local context. Knowledge translation (KT) is a well-recognized process for bringing research findings into clinical practices; one such framework, Knowledge to Action (KtA), is being used to inform the design, implementation and monitoring process of novel EEI [1]. For health professions education (HPE) to harness the advantages of EEI, researchers can draw on frameworks like the KtA to explore whether they are useful in helping tailor EEI implementation to the needs of the local environment.

HPE scholars have long advocated the adoption of EEI built on firm theoretical foundations [2–5]. The implementation of EEI should be tailored to the specificities of the local contexts to optimally support educational practices and policies, and improve learner outcomes [1, 6, 7]. Adapting EEIs involves identifying and understanding the myriad contextual factors that may influence uptake [1, 4, 6–8] including: access to and time to review the scientific evidence; positive attitudes toward the intervention; involvement in knowledge creation; and quality of available evidence [4, 9]. Unfortunately, such contextually adapted implementations are not always realized in HPE practices [8].

KT offers a structured approach to documenting contextual factors that should be considered when designing and implementing an intervention for a local context. The applicability of KT has been documented in HPE [4, 6, 7, 10], but to date, KT is seldom used to inform and improve the implementation of EEI. We propose that KT processes could support HPE’s creation and uptake of contextually adapted EEI. The purpose of this study was to design, implement and monitor an EEI guided by the KtA framework. In this manuscript we describe the implementation process for others to consider when designing, implementing, and monitoring EEI tailored to their local contexts.

## Methods

### Context

This study focuses on a longitudinal learning activity aimed at supporting the development of clinical reasoning among medical students. This activity was designed and implemented in a new four-year competency-based undergraduate curriculum at the University de Sherbrooke, Québec, Canada.

When the project reported in this paper was designed and launched, the new curriculum had been approved and the planning committee was in the process of constructing the teaching and learning activities. The first author (MC), a faculty member with expertise in clinical reasoning, joined the curriculum planning committee to consider how a newly developed EEI could be integrated into the new curriculum to support students’ development of clinical reasoning skills.

### Conceptual framework for the implementation

The KtA is a process framework designed to support the uptake of research-based knowledge into practice [11]. It consists of several steps that can guide educators in the implementation of an EEI. KtA consists of two components: knowledge creation and knowledge application (action cycle). The seven steps of the action cycle are: 1) identify the know-do gap (the gap between research and practice) and review/select relevant research-based knowledge; 2) adapt this knowledge to local context; 3) assess barriers/facilitators to knowledge use; 4) select, tailor, and implement the intervention; 5) monitor knowledge use; 6) evaluate outcomes; and 7) sustain knowledge use. Within this framework and its different steps, the term *knowledge* refers to research knowledge adapted to the context which, in our case, was the EEI.

The action cycle is dynamic and iterative. For instance, steps 3 and 4 may be repeated until the intervention is sufficiently customized to contextual specificities and users’ needs. Furthermore, the boundaries between the creation and application of knowledge are fluid—i.e., as new knowledge is created, it can inform the action cycle, and as new knowledge is implemented, teams can collect data on the implementation process, and can contribute to further refining existing knowledge or creating new knowledge. In this way, knowledge creation and knowledge application interact with and inform each other.

### EEI development and KtA process

We now present how the EEI was developed following the first five steps of the KtA framework. Because we chose to assess barriers and facilitators iteratively and throughout the implementation process, we will describe Step 4 before Step 3 below. The ultimate goal of the implementation process is to design, implement, and assess the effectiveness of a longitudinal educational activity that can support the development of students’ clinical reasoning skills and build on and align with other planned teaching/learning activities.

This study was approved by our institution’s Education—Social Sciences, Research Ethics Board (Comité d’éthique de la recherche—Éducation et sciences sociales) (protocol number: 2017–1488). All participants consented to participate.

#### KtA Step 1. Identify the know-do gap and review/select relevant research-based knowledge:

The gap to be addressed concerned the development of clinical reasoning skills in preclinical medical students. We began by reviewing the clinical reasoning literature and selecting relevant research knowledge on the topic. Drawing from this literature and the team’s content expertise, we conceptualised clinical reasoning using a cognitive perspective based on Schmidt’s theory of expertise in medicine [12, 13]. This theory describes transitory stages of medical knowledge development in medical students; it suggests that helping students progressively build deep, interconnected, coherent knowledge organized around illness scripts is essential for the development of their clinical reasoning skills. We singled out two educational interventions that have been successfully used to develop clinical reasoning: self-explanation (SE) and structured reflection (SR) [14–16]. These interventions, their rationale and the research behind them are detailed elsewhere [17–19]. These interventions target learners’ knowledge building in two different ways. In SE, learners work individually and independently through learning materials and explicitly develop and report oral explanations that deepen understanding [14, 20]. In SR, students compare and contrast plausible diagnoses for clinical cases to refine illness scripts stored in each student’s memory [15, 21].

The EEI that we implemented combined SE and SR in a longitudinal activity. A full description of the SE-SR activity has been published elsewhere [21].

#### KtoA Step 2. Adapt knowledge to local context—Transforming research based knowledge into EEI:

To implement this EEI, we needed to ensure that the evidence for SE and SR upon which the innovation is based was applicable to our local context. Specifically, we had to address the following contextual factors: the large number of students participating in the activity (i.e., a cohort of 206 students); the distributed nature of the medical program (i.e., situated in three geographically distant sites); the skill levels of the learners; and the limited availability of faculty members.

We also designed an activity that was aligned with the other characteristics of the new curriculum structured around professional clinical situations of increasing complexity; with successive blocks of small-group learning sessions through which students acquired biomedical and clinical knowledge, history and physical examination skills, problem management knowledge relevant to the clinical situations; and recurrent integration weeks that provide students the opportunity to deepen and apply their knowledge [21].

#### KtoA Step 4. Select, tailor, and implement the EEI:

Clinical teachers created the clinical cases for the SE-SR activity; each case was reviewed by the educators and curriculum planners responsible for the SE-SR activity to ensure that the case aligned with the block’s curriculum and the SE-SR activity’s delivery format. To deliver the SE-SR activity, cases were loaded onto a web platform already used by the program. The platform enabled students to access and complete the SE-SR activity individually at any time during the integration week. The platform archived each student’s work by audio recording their verbal SE and saving their written SR. Training material about clinical reasoning, SE and SR and how to engage in SE and SR via the platform was created and added to the platform. This training material, the platform, and procedures were pilot tested with volunteer students from the previous academic year. The SE-SR activity was implemented as a mandatory part of the curriculum for all students in all three sites in October 2017.

The resulting adapted educational intervention—i.e., the SE-SR activity—consisted of 11 web-based 90-minute learning sessions which students individually completed within integration weeks over the first 2.5 years of the curriculum. In each session, students engaged in both SE and SR to solve three challenging clinical cases relevant to the block’s content [21].

#### KtoA Step 3. Assess barriers and facilitators to the uptake of intervention:

Assessing the SE-SR activity’s implementation in an ongoing manner allowed for continuous refinement of the activity and maintained buy-in from learners and other stakeholders, who were involved in the design and implementation of the EEI. To that end, through biannual discussions we collected data on factors that could support or impede the implementation and uptake of the EEI. Focus groups were held with learners who were actively using the SE-SR activity, and focus groups and individual interviews were held with stakeholders (i.e., decision-makers such as educators, administrators, and vice-dean; teachers involved in creating the learning materials [e.g., SR and SE cases, training videos, etc.]; web-platform designers; and curriculum coordinators). These data supported early identification of specific problems to be addressed to improve the intervention or its uptake. These findings also informed iterations across steps 3, 4, and 5 (see below).

#### KtoA Step 5. Monitor students’ uptake of the intervention:

To track students’ engagement with the SE-SR activity, we collected quantitative data (i.e. access to SE, access to SR, time spent on the activity) from the web platform. Eighty-five percent (*n* = 175) of the class consented to anonymous data collection via the web platform. During the study period, two students dropped out of the program, five had to repeat a year, and eleven took a leave of absence.

#### Iteration across steps 3, 4, and 5. Refinement of the intervention:

Data collected from steps 4 and 5 informed iterative refinements of the SE-SR activity. Additional data were solicited via just-in-time questionnaires distributed on the platform. The questionnaire items changed at each time point (i.e., mid and end of Year 1; mid and end of Year 2) in response to the adjustments made to the activity based on the previous time point data. Refinement of the intervention was based on feedback received from both stakeholders and students. The changes were validated by the educators and curriculum coordinators. These were mostly technical: for example, increased amount of time to complete a case; moment for providing feedback; longer period of time to access the activity on the platform; change in audio-recording method.

### Assessment of the EEI’s implementation

To study the KtA implementation process and examine its success, we used a mixed-methods concurrent triangulation design [22]. In this model, qualitative and quantitative data are collected and analyzed separately, and then converged by comparing and contrasting the results during interpretation [22]. The quantitative and the qualitative data were collected at different—and sometimes overlapping—time points across 11 activities (see Appendix A of the Electronic Supplementary Material).

### Quantitative data: platform data

The SE-SR activity’s web platform collected the number of cases completed and time spent on each case. A research assistant extracted these anonymous data from the web platform at each time point. Descriptive statistics were computed to report on the fidelity and feasibility of the activity.

### Qualitative data: student and stakeholder discussions

All first-year students (*n* = 206) were invited to participate in focus group discussions at two points in each year: mid year and end of the year. The protocol for student focus groups sought overall impressions of the activity; the barriers and facilitators to its implementation; whether or not (if yes, how) students changed the way they did the activity from one time to the next; whether or not (if yes, how) the strategies of the learning activity have transferred to other contexts; whether or not (if yes, how) the activity could be improved; and whether or not (if yes, how) the activity fostered the development of clinical reasoning. Five students participated in the first focus group (i.e., mid Year 1). Twenty-four students consented for the second (i.e., end Year 1) resulting in convening three focus groups consisting of eight participants at this time point. In Year 2, we recruited 15 participants divided in two focus groups for mid Year 2, and 11 participants in two focus groups at the end of Year 2.

Stakeholders (*n* = 15) also participated in focus groups. Stakeholders were individuals who played a role in SE-SR conception and implementation. For the stakeholders we aimed to seek impressions of the activity; barriers and facilitators to its implementation; whether (if yes, how) the activity could be improved; and whether or not (if yes, how) the activity fostered the development of clinical reasoning in learners. Because of scheduling difficulties, the first (i.e., mid Year 1) stakeholder focus group was transformed into three individual interviews. We recruited five stakeholders for the second focus group (i.e., end Year 1), and four for the third focus group (i.e., mid Year 3). For the last discussion (i.e., end Year 2), again because of scheduling difficulties, we conducted four individual interviews as well as a simultaneous interview with another two stakeholders.

All focus groups and/or individual interviews were facilitated by an experienced research assistant uninvolved in the program, were audio recorded, transcribed, and anonymized. We engaged in thematic analysis [23] of the data to identify and describe barriers and facilitators. One team member (LB) conducted the initial coding process that involved minimal interpretation or abstraction of the data. This analysis aimed only to bring participant comments with similar content together into codes. These codes were then reviewed by a second team member (MC). Discussion between LB and MC led to consensus on the coding structure. A third member of the research team (AT) revised the codes and suggested elaboration, refinement, and extended several descriptions of the codes. A subsequent meeting with LB, MC, and AT led to a final coding structure which was applied to the entire dataset. The final coding structure was presented to the team for discussion and refinement of the themes (see Codebook in the Electronic Supplementary Material).

To examine the success of the implementation of this intervention, we focused on implementation outcomes. Specifically, we focused on six of Proctor et al.’s [24] implementation outcomes that, while designed for clinical settings, are equally relevant to our educational context:

- *Fidelity*: the alignment between the intervention’s actual implementation and its original intention;
- *Feasibility*: the extent to which the intervention can be successfully used in the program;
- *Appropriateness*: the perceived fit of the intervention for the program;
- *Acceptability*: the perception of stakeholders that the intervention is satisfactory;
- *Adoption*: the intention by the organization and the providers to employ the intervention;
- *Penetration*: the integration of the intervention in the program.

Quantitative and qualitative data were integrated and aligned with these outcomes measures. Table 1 lists which data were used as evidence for each of the six outcomes.

**Table 1:** Outcomes and data alignment

Outcomes	Data
Fidelity	Students’ mean total time spent doing the activity
Feasibility	Students’ completion rate for each case Students’ verbatim Stakeholders’ verbatim
Appropriateness	Students’ verbatim Stakeholders’ verbatim
Acceptability	Students’ verbatim Stakeholders’ verbatim
Adoption	Stakeholders’ verbatim
Penetration	Students’ verbatim Stakeholders’ verbatim

## Results: outcomes

### Fidelity and feasibility

Mean total time spent on the activity, presented in Tab. 2, informed our understanding of the *fidelity* (students’ time spent on the learning activity) of the implementation. Furthermore, the completion rate for each case provided insights into the *feasibility* of the implementation (percentage of students doing the activity). Throughout cases 1–21, technical problems with the platform’s audio recording occurred at random; therefore, not all SE-SR recordings were saved resulting in incomplete data on SE for some students. The lower number of SE-SR completed in cases 4, 5, and 6 occurred because the Program Directors decided, just for cases 4–6, to require students to complete only one case of their choice among the three case options—a change made in response to students’ reported work overload at that point in the new curriculum. Excluding cases 4–6, the mean completion rate of SE and SR for cases 1 to 21 was 81%, while the mean completion rate from case 22 to 33 (after the technical change in the recording process) was 92%. Students spent an overall mean time of 24:08 min (SD = 2:07 min, range 19:52–28:28 min) for each case.

**Table 2:** Frequencies of SE-SR completed and mean total time spent on each case

Activity	Cases	N	Number of SE-SR completed (%)	Mean total time spent on a case (SD)
1	1	175	150 (86)	25:38 (4:43)
	2	175	156 (89)	26:31 (4:06)
	3	175	158 (90)	23:14 (4:48)
2	4	174	92 (53)	28:28 (5:29)
	5	174	73 (42)	28:06 (6:15)
	6	174	44 (25)	28:28 (6:07)
3	7	173	131 (76)	24:32 (6:23)
	8	173	141 (82)	22:34 (6:19)
	9	173	140 (81)	23:11 (6:43)
4	10	173	145 (84)	24:26 (6:16)
	11	173	145 (84)	25:46 (6:40)
	12	173	141 (82)	24:51 (6:16)
5	13	172	131 (76)	26:08 (6:35)
	14	172	134 (78)	22:11 (6:32)
	15	172	136 (79)	24:16 (6:30)
6	16	167	125 (75)	21:32 (5:45)
	17	167	132 (79)	20:01 (6:20)
	18	167	128 (77)	24:11 (6:29)
7	19	167	134 (80)	22:51 (6:03)
	20	167	127 (76)	23:58 (6:03)
	21	167	131 (78)	19:52 (5:43)
8	22	167	152 (91)	24:48 (6:06)
	23	167	149 (89)	25:41 (6:20)
	24	167	149 (89)	22:54 (6:24)
9	25	167	158 (95)	26:12 (7:01)
	26	167	157 (94)	24:32 (6:19)
	27	167	156 (93)	21:56 (6:31)
10	28	166	157 (95)	22:58 (6:41)
	29	166	156 (94)	22:19 (6:27)
	30	166	155 (93)	23:48 (7:03)
11	31	157	143 (91)	24:39 (7:01)
	32	157	144 (92)	22:16 (6:39)
	33	157	143 (91)	23:38 (7:15)

### Feasibility

Stakeholders reported that the activity was easy to run after its implementation, once the platform was set up. They reported that it required no more effort than monitoring the platform’s use and bringing adjustments when needed.

### Appropriateness

Students reported many ways in which the activity was relevant and appropriate for their learning. Students perceived that SE-SR helped them monitor their knowledge and guide their study; it was like practicing for exams. They also perceived the activity as preparation for future clinical work. Stakeholders perceived the activity as aligned with the program and its objective, i.e., to develop clinical reasoning skills early in the curriculum.

### Acceptability

Students and stakeholders perceived the activity as enjoyable. As one student said: “*When you just apply your knowledge it’s a lot of fun. It’s like playing around with your knowledge, trying to find the diagnosis, the differential. It’s more fun.*” (FG1-Students-Jan 2019). A stakeholder expressed it with: “*It’s a good activity […] that seems useful and not too labour-intensive.”* (Int6-Stakeholders-June 2019).

### Adoption

Stakeholders involved in setting up the activity viewed the activity in a positive light and were keen to participate in the conception, as expressed in this quote: “*It’s got very good buy-in from the program members, not just the development committee, but also of the coordinators of the other activities along with the designers who made up the clinical vignettes*.” (Int1-Stakeholder-Jan 2018)

### Penetration

Students explained how, over time, doing the activity became a habit and thus easier. Stakeholders recognized that, as a long-term activity, SE-SR would become easier for students and they would grasp its potential; as such, it would be beneficial for students to become acquainted with the activity. They found that the activity was well integrated into the curriculum.

## Discussion

Our results suggest that the implementation of the EEI was successful, in terms of fidelity, feasibility, appropriateness, acceptability, adoption, and penetration.

Though we cannot report how much of the success was due to our KT efforts which were guided by the KtA framework, we share three main lessons learned from this implementation process and offer possible reasons why it may have contributed to the success.

### Lesson one: Knowledge translation frameworks and methods

Using a well-known KT framework (i.e., KtA) and robust methods can help guide implementation research [25–27]. Using the KtA framework allowed us to move research evidence on medical students’ clinical reasoning development into practice (i.e., into the curriculum) in a deliberate manner. Doing so required outlining the stages of the research-to-practice translation processes all the way from production of the research-based knowledge to its implementation and use in a specific context [26].

The KtA framework guided the implementation process by helping us to systematically and iteratively provide a strong rationale for ongoing adaptation of the activity and its implementation. Data on barriers and facilitators allowed the implementation team to make timely data-driven modifications to the intervention thereby ensuring its continued relevance and applicability in this program. Furthermore, the use of quantitative and qualitative data had several benefits—most notably it enabled us to adjust appropriately and quickly the intervention in response to feedback. The KtA framework was therefore ideally suited for our purposes.

Despite such benefits, using the KtA framework also created challenges. Most notably, adhering to each step of KtA was time consuming and slowed the implementation process; required much pre-planning and buy-in from various stakeholders; and necessitated that the implementation team be well versed in the KtA to convince students and stakeholders—as to why it was necessary. Though we work in a context where these challenges could be overcome, we acknowledge that this may not be possible in all settings. We propose that implementation teams discuss the usefulness of such deliberate approaches early in the process to ensure that the methods used and the outcomes generated align with the values, priorities, and resources of the local context [1, 8].

### Lesson two: Diversity of perspectives and collaborative approach

Our team consisted of a combination of clinicians, curriculum designers, educators, HPE researchers, and an expert in KT. Having representation and expertise from each of these groups was key to planning and executing this implementation project in an authentic practice context [28, 29]. Building on this expertise we adopted a collaborative approach with two groups of stakeholders: local decision-makers and students.

Buy-in from local decision-makers is more likely to result in knowledge that is relevant for, and valuable to the program and, ultimately, be used to bring about meaningful change in program and learner outcomes.

Though student involvement in curriculum planning and delivery is not uncommon in HPE, implementation efforts are often predicated upon multi-stakeholder involvement rather than concentrating on students’ feedback [30]. We underline the importance of student participation. Through it, learners were kept collectively aware that their feedback would be used at regular intervals to generate changes in the educational intervention. They could then witness these changes in real-time. For successful student participation in an iterative, longitudinal implementation process, care must be taken in choosing the times when they are asked to provide input, and in selecting the most useful types of data collection. This can avoid over-solicitation of learners, especially in a context of curriculum renewal when they are frequently invited to contribute to many different activities. HPE programs should think about the conditions necessary to ensure optimal and authentic learner participation [31] in implementation projects.

### Lesson three: Concurrent implementation and evaluation

Concurrent implementation (KtA Step 3), assessment (KtA Step 4) and monitoring (KtA Step 5) likely contributed to the success of our EEI implementation. Although the KtA framework presents these phases sequentially, our experience suggests that authentic practice environments greatly benefit from these phases occurring simultaneously. Indeed, KtA scholars suggest that a more fluid and flexible approach to the seven stages is best to contend with the constraints of individual contexts [11]. A concurrent approach, such as the one in this project, helps implementation teams to respond in a timely manner by adjusting each aspect of the implementation in response to stakeholder feedback. Importantly, this strategy may garner additional buy-in from decision makers who require evidence of implementation success when advocating for and allocating resources for such substantial curricular changes.

### Limitations

This study was conducted in a specific context and its applicability to other contexts may be limited. However, our purpose was to illustrate the implementation of an EEI using a KT framework. Therefore, we hope that our explanations and illustrations of this process is something that can transfer to other contexts.

One may argue that the fact that the amount of time spent on the activity by students corresponds to what was planned and expected may be only an indirect indicator of fidelity. However, in additional studies, we assessed directly the quality of students SE audio recordings and written SR and could observe that students were engaged in these strategies as intended [32, 33].

Furthermore, limitations inherent to focus groups, interviews and web platform data place a constraint on the insights and interpretations we can generate from our data. For instance, the small pool of students who volunteered for the focus groups is unlikely to be representative of the entire student body; however, we aimed to partially circumvent this limitation by collecting quantitative data from the whole class via the web platform. No apparent discrepancy was noted between the qualitative and quantitative student data, but it remains possible that alternative viewpoints impacting on our perception of the implementation and on the process of implementation itself were missed because these viewpoints were not expressed by the recruited students.

## Conclusion

We have presented the KtA-informed implementation of an EEI in the setting of a curriculum renewal in a Canadian medical undergraduate program. The KtA approach offered a structured yet flexible approach to designing, implementing, and monitoring an EEI tailored to our local contexts. Future work could focus on generating evidence of its applicability in a variety of HPE contexts and measuring multilevel outcomes.

## Supplementary Information

Appendix A

Codebook

## References

[1] Thomas A, Bussières A (2021). Leveraging knowledge translation and implementation science in the pursuit of evidence informed health professions education. Adv Health Sci Educ.

[2] Durning SJ, Dolmans DHJM, Cleland J, Mennin S, Amin Z, Gibbs TJ (2012). The AMEE Research Committee: Initiatives to stimulate research and practice. Med Teach.

[3] van der Vleuten CPM, Dolmans DHJM, Scherpbier AJJA (2000). The need for evidence in education. Med Teach.

[4] Thomas A, Gruppen LD, van der Vleuten C, Chilingaryan G, Amari F, Steinert Y (2019). Use of evidence in health professions education: attitudes, practices, barriers and supports. Med Teach.

[5] Dauphinee WD, Wood-Dauphinee S (2004). The need for evidence in medical education: the development of best evidence medical education as an opportunity to inform, guide, and sustain medical education research. Acad Med.

[6] Thomas A, Bussières A (2016). Knowledge translation and implementation science in health professions education: time for clarity?. Acad Med.

[7] Thomas A, Bussières A (2016). Towards a greater understanding of implementation science in health professions education. Acad Med.

[8] Thomas A, Ellaway RH (2021). Rethinking implementation science for health professions education: a manifesto for change. Perspect Med Educ.

[9] Onyura B, Légaré F, Baker L (2015). Affordances of knowledge translation in medical education: a qualitative exploration of empirical knowledge use among medical educators. Acad Med.

[10] Tractenberg RE, Gordon M (2017). Supporting evidence-informed teaching in biomedical and health professions education through knowledge translation: an interdisciplinary literature review. Teach Learn Med.

[11] Graham ID, Logan J, Harrison MB (2006). Lost in knowledge translation: time for a map?. J Contin Educ Health Prof.

[12] Schmidt HG, Rikers RMJP (2007). How expertise develops in medicine: knowledge encapsulation and illness script formation. Med Educ.

[13] Schmidt HG, Norman GG, Boshuizen HH (1990). A cognitive perspective on medical expertise: theory and implications. Acad Med.

[14] Chamberland M, St-Onge C, Setrakian J (2011). The influence of medical students’ self-explanations on diagnostic performance. Med Educ.

[15] Mamede S, van Gog T, Moura AS (2012). Reflection as a strategy to foster medical students’ acquisition of diagnostic competence. Med Educ.

[16] Schmidt HG, Mamede S (2015). How to improve the teaching of clinical reasoning: a narrative review and a proposal. Med Educ.

[17] Chamberland M, Mamede S (2015). Self-explanation, an instructional strategy to foster clinical reasoning in medical students. Health Prof Educ.

[18] Mamede S, Schmidt HG (2022). Deliberate reflection and clinical reasoning: founding ideas and empirical findings. Med Educ.

[19] Torre D, Chamberland M, Mamede S (2022). Implementation of three knowledge-oriented instructional strategies to teach clinical reasoning: Self-explanation, a concept mapping exercise, and deliberate reflection: AMEE Guide. Med Teach.

[20] Chi MTH, Glaser R (2000). Self-explaining expository texts: The dual processes of generating inferences and repairing mental models. Advances in instructional psychology.

[21] Chamberland M, Mamede S, Bergeron L, Varpio L (2021). A layered analysis of self-explanation and structured reflection to support clinical reasoning in medical students. Perspect Med Educ.

[22] Creswell JW, Plano CVL, Gutmann ML, Hanson WE, Tashakkori A, Teddlie C (2003). Advanced mixed methods research designs. Handbook of mixed methods in social and behavioral research.

[23] Kiger ME, Varpio L (2020). Thematic analysis of qualitative data: AMEE guide No. 131. Med Teach.

[24] Proctor E, Silmere H, Raghavan R (2011). Outcomes for implementation research: conceptual distinctions, measurement challenges, and research agenda. Adm Policy Ment Health.

[25] Birken SA, Powell BJ, Shea CM (2017). Criteria for selecting implementation science theories and frameworks: results from an international survey. Implement Sci.

[26] Nilsen P (2015). Making sense of implementation theories, models and frameworks. Implement Sci.

[27] Milat AJ, Li B (2017). Narrative review of frameworks for translating research evidence into policy and practice. Public Health Res Pract.

[28] Boaz A, Hanney S, Borst R, O’Shea A, Kok M (2018). How to engage stakeholders in research: design principles to support improvement. Health Res Policy Syst.

[29] Goodman MS, Sanders Thompson VL (2017). The science of stakeholder engagement in research: classification, implementation, and evaluation. Transl Behav Med.

[30] Huppatz C (1996). The essential role of the student in curriculum planning. Med Educ.

[31] Thomas A, Kuper A, Chin-Yee B, Park M (2020). What is “shared” in shared decision-making? Philosophical perspectives, epistemic justice, and implications for health professions education. J Eval Clin Pract.

[32] Chamberland M, Setrakian J, Marceau M (2020). Validation of a grid to document the quality of self-explanation when implemented as a learning strategy at the UGME level. Presented at CCME 2020 (Canadian Conference on Medical Education). Can Med Educ J.

[33] Chamberland M, Setrakian J, Vachon Lachiver E (2020). Validation of a grid to document the quality of structured reflection when implemented as a learning strategy at the UGME level. Presented at CCME 2020 (Canadian Conference on Medical Education). Can Med Educ J.

