# Supplementary material for: Harnessing a knowledge translation framework to implement an undergraduate medical education intervention: A longitudinal study
Source: Perspect Med Educ. 2022 Dec 7;11(6):333–40. doi: 10.1007/s40037-022-00735-7 (PMC9743946; doi:10.1007/s40037-022-00735-7)
Supplement: Supplementary file 1 — Appendix A [file 40037_2022_735_MOESM1_ESM.docx]

**Appendix A**

Chronology of the implementation of SE-SR activity, and concurrent data collection

| Year | | Year 1 | | | | | Year 2 | | | | | Year 3 | |
| --- | --- | --- | --- | --- | --- | --- | --- | --- | --- | --- | --- | --- | --- |
| Activity | Activity number | Act 1 | Act 2 | Act 3 | Act 4 | Act 5 | Act 6 | Act 7 | Act 8 | Act 9 | Act 10 | Act 11 |  |
|  | Clinical cases numbers | 1-3 | 4-6 | 7-9 | 10-12 | 13-15 | 16-18 | 19-21 | 22-24 | 25-27 | 28-30 | 31-33 |  |
| Data collection | Plateform data (n) | x | x | x | x | x | x | x | x | x | x | x |  |
|  | Focus group and/or individual interviews |  |  |  |  |  |  |  |  |  |  |  |  |
|  | Stakeholders |  | x |  |  | x |  | x |  |  | x |  |  |
|  | Students |  | x |  |  | x |  | x |  |  | x |  |  |
