# Supplementary material for: Harnessing a knowledge translation framework to implement an undergraduate medical education intervention: A longitudinal study
Source: Perspect Med Educ. 2022 Dec 7;11(6):333–40. doi: 10.1007/s40037-022-00735-7 (PMC9743946; doi:10.1007/s40037-022-00735-7)
Supplement: Supplementary file 2 — Codebook [file 40037_2022_735_MOESM2_ESM.docx]

| **Barriers/**  **facilitators** | **Category** | **Codes** | **Descriptions** | **Number of exerpts** | **Sources** |
| --- | --- | --- | --- | --- | --- |
| Barrier | Activity | Some case content not aligned with the preceeding learning block contents | Clinical cases have never been used before this first cohort of medical students. This first year highlights some misalignments in the new material. When the cases are less connected to what has been seen in class, students perceive the usefulness of the activity less well and may experience stress about whether they should know this information for their current purposes. | 25 | Students and stakeholders |
|  |  |  |  |  |  |
| Barrier | Activity | Early on, completed SR grid available only at the end of the period | The moment when students receive the answer key (feedback) is too late. | 5 | Stakeholders |
| Barrier | Activity | Assessment, not of contents produced by students, but only of the process is perceived as a problem by some professors. | The fact that the activity is not assessed means that some students may engage less seriously with the task. | 3 | Stakeholders |
| Barrier | Activity | The timing of the activity is problematic for some students | Time slot limited to three days initially: this does not allow to have in one’s memory the knowledge needed to perform the activity and to perceive it as useful. Also, competition with study period before exams: internal conflict between time and priority management. | 45 | Students and stakeholders |
|  |  |  |  |  |  |
| Facilitator | Activity | Material intended for students to get familiar with the activity | Usefulness of practice (being able to do a case that doesn't count), and instructions to know how it works and take the stress out of seeing what's coming. Moreover, the value of the activity is well explained to the students through the material presented. | 52 | Students and stakeholders |
|  |  |  |  |  |  |
|  |  |  |  |  |  |
|  |  |  |  |  |  |
| Facilitator | Activity | Quality of the clinical cases | The clinical cases of the activity are well designed. They are perceived as a way to reactivate knowledge or to expand one's reasoning. May even allow students to see things that were not seen in class. | 25 | Students and stakeholders |
|  |  |  |  |  |  |
| Facilitator | Activity | Technical platform (record capabilities, subsequent access to audio-recording, easy to use) | The web platform used to contain the activity web-based allows us to offer the activity online with the characteristics we want it to have (time constraint, audio recording, etc.). For those students who did not have technology issues, the activity was easily accessible, and everything worked well. Recording the SE and SR in the student's file allows the student to return to it. | 19 | Students and stakeholders |
|  |  |  |  |  |  |
|  |  |  |  |  |  |
|  |  |  |  |  |  |
| Facilitator | Activity | Completed SR grid perceived to be useful, even essential. | The answer key is seen as essential to the activity. Students appreciate receiving feedback right away. Students used the answer key of the SR grid to study. | 39 | Students |
|  |  |  |  |  |  |
| Facilitator | Activity | Low-stake activity | The formative aspect of the activity (as opposed to summative) helps the student feel more comfortable doing the activity and removes stress. It also facilitates students’ buy-in and avoids problems related to cheating. | 30 | Students and stakeholders |
|  |  |  |  |  |  |
| Facilitator | Activity | Recurring activity | Repeating the activity allows the student: - to understand better what is expected of them because of the feedback they received or because they did it the first time and therefore know how it will go. - to have dealt with the technology issues. - to become more comfortable with the method. - to develop strategies that pay off. | 30 | Students and stakeholders |
|  |  |  |  |  |  |
|  |  |  |  |  |  |
|  |  |  |  |  |  |
| Facilitator | Activity | Compulsory activity | Some see optionality would lead some students to leave out doing some activities and thereby missing out on something that could be useful for their learning. Stakeholders also think that students may be more inclined to participate in the activity because it is mandatory. | 15 | Students and stakeholders |
|  |  |  |  |  |  |
|  |  |  |  |  |  |
| Facilitator | Activity | Facilitating instruction related to SE | Verbalizing aloud and recording oneself, plus having an instruction of doing SE for a minimum time of six minutes encourage students to be invested in their SE. | 12 | Students and stakeholders |
|  |  |  |  |  |  |
| Facilitator | Activity | Having a time limit to complete each case, while allowing enough time to complete the activity | The fact that the activity should not take more than 35 minutes per case is seen as positive to help not waste too much time and be more efficient. | 26 | Students and stakeholders |
|  |  |  |  |  |  |
|  |  |  |  |  |  |
| Facilitator | Activity | Timing of the activity: Up to the student to choose the exact moment of performing the activity within a set time slot (flexibility) | The flexibility of when to do the activity is appreciated by students. The timing of the activity in the students' schedule is optimal. | 27 | Students and stakeholders |
|  |  |  |  |  |  |
| Facilitator | Activity | Cases can be done separately and in the chosen order. | Students really appreciate knowing what the case is going to be about. They can prepare, have the right diagnoses in mind, and better choose when to do the case. | 22 | Students |
|  |  |  |  |  |  |
| Barrier | Resources and context | Students’ work overload | The activity is implemented in a context of a complete curriculum reform and students feel overloaded. First-year students for whom everything is new are given a schedule that often changes, and several activities are added as the session unfolds. If students are stuck in time for their study, they will choose not to do the activity (we see this among other things when they were given the choice to do all three cases or not). | 56 | Students and stakeholders |
|  |  |  |  |  |  |
|  |  |  |  |  |  |
|  |  |  |  |  |  |
| Barrier | Resources and context | Administrative overload of the new curriculum | Lack of time to see things through and plan what needs to be done given the overload of work in the new curriculum. | 4 | Stakeholders |
|  |  |  |  |  |  |
| Barrier | Resources and context | Multiple and erratic communications | Communication of the program to students difficult: there is repetition, but the information is diverse, and it is not clear or the information is not timely. This leads to a negative perception toward new activities. | 11 | Students and stakeholders |
|  |  |  |  |  |  |
| Barrier | Resources and context | Lack of support by IS (at the Faculty/University level) for some technical problems | Support by IS does not provide the necessary assistance for the smooth running of the activity in terms of decisions to be made and technical support to solve problems. | 19 | Stakeholders |
| Barrier | Resources and context | Isolated technical problems (difficulties with audio recording, user errors, display issues) | A lack of proficiency with the technologies can cause stress for students when doing the educational activity. Moreover, it is difficult to predict all possible computer problems, and student may need to consult all available resources to assist in the completion of the activity by lack of interest or time. This prevents students from getting the help they need to complete the activity. | 79 | Students and stakeholders |
|  |  |  |  |  |  |
|  |  |  |  |  |  |
|  |  |  |  |  |  |
|  |  |  |  |  |  |
|  |  |  |  |  |  |
|  |  |  |  |  |  |
|  |  |  |  |  |  |
| Facilitator | Resources and context | Undergraduate program taking back control over the audio recording server | It was facilitating to have the simplest possible process to access the activity. The platform where the activity is located has been modified so that it is more easily accessible. For example, the way in which the self-explanation is recorded for the activity has been changed, eliminating the difficulty of audio recording. | 9 | Students and stakeholders |
|  |  |  |  |  |  |
|  |  |  |  |  |  |
| Facilitator | Resources and context | Support offered to students to facilitate the access to the activity | Several tests were done before the activity began to find solutions and create procedures to help students during the activity. Resources were available to students to facilitate access to, and completion of, the activity. Use of techno "super-user" students (already identified for all techno aspects of the program) who assist their peers as needed and who have been actively involved in developing and implementing a recording alternative for SE. | 27 | Students and stakeholders |
|  |  |  |  |  |  |
|  |  |  |  |  |  |
|  |  |  |  |  |  |
| Facilitator | Resources and context | Support by stakeholders | The activity is perceived positively by the stakeholders as an educational method. | 16 | Stakeholders |
| Facilitator | Resources and context | Curricular alignment | The activity was implemented based on studies that support its effectiveness in teaching clinical reasoning in medicine. The activity addresses a program need to provide a self-directed, formative learning activity. The activity fits well into the medicine program and is consistent with other program activities and exams. The cases proposed in the activity become increasingly complex, which would motivate students to engage in the process. | 27 | Students and stakeholders |
|  |  |  |  |  |  |
|  |  |  |  |  |  |
|  |  |  |  |  |  |
| Facilitator | Resources and context | Availability of case developers | Faculty have agreed to collaborate on this activity and became case developers. | 9 | Stakeholders |
|  |  |  |  |  |  |
|  |  |  |  |  |  |
| Facilitator | Resources and context | Requires little commitment on the part of faculty members | Once set up, this activity costs almost nothing to run. | 11 | Stakeholders |
|  |  |  |  |  |  |
| Facilitator | Resources and context | Well structured process by the design team | Implementing the activity in the program required (among other things) activity leaders and program leaders. Choices about the activity were made following discussions between stakeholders: the program and the activity designers. It was easy to get access to the person responsible for the activity to discuss adjustments to be made. | 14 | Stakeholders |
|  |  |  |  |  |  |
|  |  |  |  |  |  |
|  |  |  |  |  |  |
|  |  |  |  |  |  |
| Facilitator | Resources and context | Adjustments brought to the activity during implementation | By monitoring the activity as it unfolds, we encourage a reaction/response to the students’ requests or needs for the smooth running of the activity. Students perceive, through the feedback given following the first activity, that they are supported in doing this activity. | 25 | Stakeholders |
|  |  |  |  |  |  |
| Barrier | Students | Performance-related stress for students | Students may lack confidence at first to do the activity well. | 7 | Students |
| Barrier | Students | Misunderstanding of the activity by some students. | Some students did not understand some of the instructions for the activity, its purpose, or what was happening with the audios they were recording. Some students find writing in the SR table redundant to what they just said in the SE. This may cause some stress or cause students not to engage in all parts of the activity correctly. | 29 | Students |
|  |  |  |  |  |  |
| Facilitator | Students | Perceived value of the activity:  Preparation for future practice through case exposure (that allows to build, expand, integrate, and deepen knowledge). | Students see the activity as a way to prepare their minds to think in a certain way, as will be required of them later, for example in an internship. The activity is perceived as a way to reactivate knowledge or to extend one's reasoning. May even allow you to see things that were not seen in class. It helps the student identify what he/she is less familiar with and therefore needs to revisit in his/her study. The activity allows the student to be exposed to cases. Doing the activity highlights gaps and provides study to fill those gaps. The activity itself is used as study time by the students. SE and SR are perceived as complementary and therefore both parts are appreciated. Students perceive the pedagogical value of doing the activity when it needs to be done. Some students prefer self-explanation which allows them to go further in their knowledge. Students appreciate the questions at the end of the activity that help them complete their resolution of the case. Some students prefer the SR grid that guides/structures them in how they solve the case. | 259 | Students and stakeholders |
|  |  |  |  |  |  |
|  |  |  |  |  |  |
|  |  |  |  |  |  |
|  |  |  |  |  |  |
|  |  |  |  |  |  |
|  |  |  |  |  |  |
|  |  |  |  |  |  |
|  |  |  |  |  |  |
|  |  |  |  |  |  |
|  |  |  |  |  |  |
|  |  |  |  |  |  |
| Facilitator | Students | Relevance of global feedback (capsules) to students on SE | Students perceive, through the feedback given following the first activity, that they are supported to do this activity. | 4 | Stakeholders |
